# Developing a comprehensive time series of GDP per capita for 210 countries from 1950 to 2015

**DOI:** 10.1186/1478-7954-10-12

**Published:** 2012-07-30

**Authors:** Spencer L James, Paul Gubbins, Christopher JL Murray, Emmanuela Gakidou

**Affiliations:** 1 Institute for Health Metrics and Evaluation, University of Washington, 2301 Fifth Ave., Suite 600, Seattle, WA, 98121, USA

**Keywords:** GDP, GDP per capita, Income, Social determinants, Covariate, Indicator

## Abstract

**Background:**

Income has been extensively studied and utilized as a determinant of health. There are several sources of income expressed as gross domestic product (GDP) per capita, but there are no time series that are complete for the years between 1950 and 2015 for the 210 countries for which data exist. It is in the interest of population health research to establish a global time series that is complete from 1950 to 2015.

**Methods:**

We collected GDP per capita estimates expressed in either constant US dollar terms or international dollar terms (corrected for purchasing power parity) from seven sources. We applied several stages of models, including ordinary least-squares regressions and mixed effects models, to complete each of the seven source series from 1950 to 2015. The three US dollar and four international dollar series were each averaged to produce two new GDP per capita series.

**Results and discussion:**

Nine complete series from 1950 to 2015 for 210 countries are available for use. These series can serve various analytical purposes and can illustrate myriad economic trends and features. The derivation of the two new series allows for researchers to avoid any series-specific biases that may exist. The modeling approach used is flexible and will allow for yearly updating as new estimates are produced by the source series.

**Conclusion:**

GDP per capita is a necessary tool in population health research, and our development and implementation of a new method has allowed for the most comprehensive known time series to date.

## Background

Income per capita is one of the most widely used socioeconomic predictors of health, and the relationship between income and health has been studied extensively. In his seminal work in 1975, Preston [1] framed three ways in which income and health are related, focusing on mortality as a measure of health. These mechanisms, summarized in the Preston curve, suggest that the level of income influences the level of health, the level of income influences the rate of change in health, and the rate of change of income influences the rate of change of health. Further economic and demographic research has also illustrated the depth of this relationship [2-9]. Gross domestic product (GDP) per capita is the most widely used indicator for country-level income [10] and has been used in modeling health outcomes [11], mortality trends [12,13], cause-specific mortality estimation [12], health system performance and finances [13,14], and several other topics of interest.

Over the years, the implications of these studies cultivated a global focus on improving health through economic policy and growth. The converse relationship, i.e., the effect of health on the economy, has also been studied extensively by macroeconomists [15-18]. In 2000, the World Health Organization (WHO) launched the Commission for Macroeconomics in Health [19], which studied the dynamics through which health impacts economic integrity. The commission heralded new goals and guidelines, which suggested that health interventions resulting in the aversion of 330 million disability-adjusted life years by 2010 would produce savings of up to US$ 180 billion per year by 2015. Later, in 2005, WHO started the Commission for Social Determinants in Health [20], which sought to develop a more comprehensive framework to describe factors that predict health and to also highlight the critical role of economic well-being in the attainment of better health.

Given the critical relationship between income and health, GDP per capita is one of the most widely used covariates in population health research. It is also one of the most regularly measured economic indicators, with estimates produced quarterly or annually by countries themselves as well as agencies such as the World Bank (WB) [21], the United Nations Statistics Division [22], and the International Monetary Fund (IMF) [23] and by institutions such as the University of Pennsylvania [24] and the University of Groningen [25]. The currently available data sources suffer from a series of limitations. First, the calculation of GDP varies across sources [26] (though it is generally defined as being the sum of private consumption, gross investment, government spending, and net exports [exports minus imports] [27]). Second, each of the sources for GDP per capita provides estimates for a range of country-years, but no particular source provides a complete dataset for all countries and years, which is often what is needed by health researchers. Third, GDP per capita estimates for any given country-year can vary dramatically depending on the source [26]. This variation is particularly exaggerated in developing countries with low economic infrastructure or unstable economic conditions [28], which are often the countries of great interest to population health researchers. Finally, geopolitical events causing a state’s acquisition or loss of sovereignty can result in extended time periods without GDP estimates. This is most evidently the case for the former Soviet Union, where no GDP estimates exist for any of the constituent republics prior to the USSR’s dissolution. This combination of issues means that any study involving the use of GDP per capita can be subject to significant variation and completeness depending on the sources used and on the country-time period of interest.

To address some of these limitations, in this paper we propose a method for achieving two goals. Goal 1 was to impute missing country-years for each available series for all countries and years from 1950 to 2015. Goal 2 was to create a new US dollar (USD) series and a new international dollar (ID) (purchasing power parity [PPP]) series based on the competed source series also comprehensive of 210 countries from 1950 to 2015.

## Methods

### Data sources

We have identified seven available GDP per capita time series. GDP per capita time series were downloaded from the University of Pennsylvania (Penn) Center for International Comparisons of Production, Income, and Prices [24], the WB World Development Indicators [21], the United Nations Statistics Division (UNSTAT) [22], the IMF World Economic Outlook report [23], and from Angus Maddison’s research homepage at the University of Groningen Department of Economics [25]. The Maddison, Penn, IMF, and WB estimates are available in ID series using PPP ratios. The UNSTAT, IMF, and a second WB series were available in constant or current USD. Thus, four series in ID and three series in USD were available. Table 1 provides details on the currencies and country-year coverage for each source series.

**Table 1:** Available data sources and time span of GDP estimates

**Series**	**Maddison**	**Penn**	**World Bank**	**IMF**	**UN**
**Current LCUs**	--	--	·	X	--
**Constant LCUs (base year)**	--	--	X (2005)	†· (varies)	--
**Current USD**	--	--	·	·	X
**Constant USD (base year)**	--	--	X (2000)	--	· (2005)
**Current ID**	--	X	X	†	--
**Constant ID (base year)**	† (1990)	† (2005)	† (2005)	--	--
**Years covered**	1950-2008	1950-2009	1960-2009	1980-2015	1970-2009

X = series exists.

† = used for international dollar series.

· = used for US dollar series.

The Penn and WB ID series were expressed in 2005 constant ID. The IMF ID series was expressed in “current” or “historical” ID and also in constant local currency units (LCUs), neither of which were immediately comparable to the other series. To convert the series to constant 2005 ID, the current ID value for the year of 2005 was kept for each country, and the growth rate derived from the constant LCU series was applied to this value to chain estimates forward and backward. This created the IMF constant 2005 ID series. The Maddison series offered portions of GDP estimates that were integral to our analytical strategy but also had two main weaknesses. First, the Maddison series was expressed only in constant 1990 ID, and since there was no reliable method for converting these to 2005 ID, we only used this series as a predictor variable in modeling the missing portions of our other series. Since the series is expressed in constant 1990 terms, this limitation should not inhibit our modeling strategy’s ability to make accurate estimations. The second weakness of the Maddison series was that it was based on the most recent set of national accounts data released no later than March 2010. Other GDP estimates were based on more recently updated sets of data. Despite this older data vintage, we opted to include the Maddison series in our analysis because it offered estimates for 8,693 country-years (out of 15,780 country-years possible), many of which were not included in the other GDP sources at our disposal. Consequently, we were able to input more data into our models, particularly for the years before 1980 and for the former Soviet Union. This implementation is described in more detail below.

**Figure 1:**
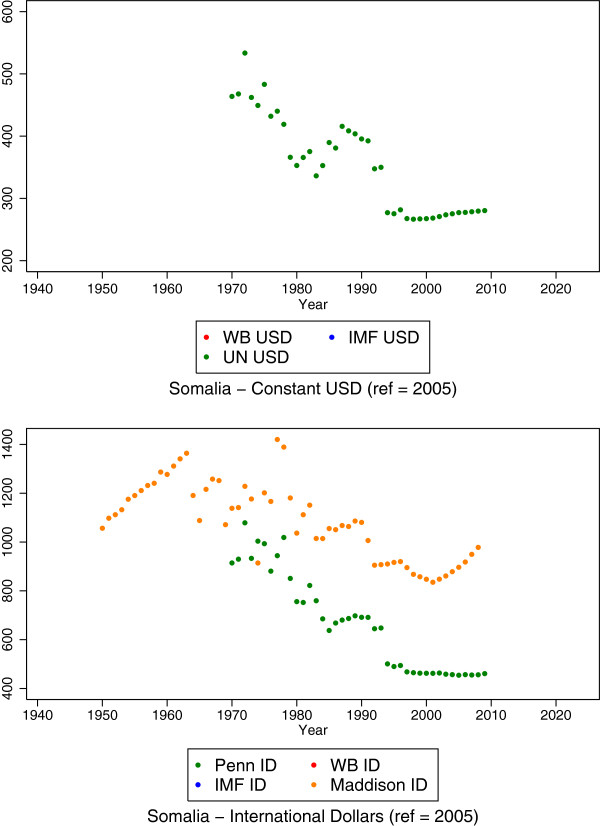
**Existing GDP per capita data for Somalia.** The World Bank and IMF series provide no estimates, while other sources provide estimates for different ranges of years.

The UNSTAT USD series was expressed in constant 2005 USD. The IMF current USD series was converted to a constant 2005 USD series using the same technique described for IMF ID above. The WB constant 2005 USD series was created by converting the WB constant 2005 LCU GDP per capita series into constant 2005 USD using 2005 exchange rates from LCU to USD. The result of this stage was three series in constant 2005 USD and four series in constant 2005 ID. Figure 1 shows the starting data in Somalia as an example. We will use Somalia to demonstrate the methods we applied to arrive at complete time series for each country. We use Somalia because the data are not complete from 1950 through 2015, there are no estimates from the World Bank and the IMF, and the existing series do not span the entire time frame. Existing data by series for Somalia are listed in Table 2. We provide the estimates that are provided or imputed at each stage of the modeling process in Additional file 1: Annex 1.

**Table 2:** Time span of available data from each source series for Somalia

**Series**	**Range of available years of data**
IMF international dollar, 2005 base year	-
Penn international dollar, 2005 base year	1970-2009
World Bank international dollar, 2005 base year	-
Maddison international dollar, 1990 base year	1950-2008
World Bank US dollar, 2005 base year	-
IMF US dollar, 2005 base year	-
UNSTAT US dollar, 2005 base year	1970-2009

During the assessment of data and development of a modeling method we observed a number of outliers. These data points seemed implausibly high or low for a particular country-series-year in the context of surrounding data points and dramatically altered the predictions from our models when included. Consequently, after confirming on a case-by-case basis that there were no outstanding geopolitical or economic incidents that could explain the anomaly, we removed the points from the series prior to modeling our estimates. Out of a total of 48,781 data points, we identified 111 as outliers. The specific country-series-year data points that were removed are listed in Additional file 2: Annex 2.

### Models

We approached Goal 1 of filling in the missing years between 1950 and 2015 for each series and predicting a series if a particular source did not include estimates for a given country through the following steps.

### Imputing missing years for existing series

We started the project with 48,670 country-year-series of data (after removing outliers and former countries such as Former Yugoslavia) distributed unevenly across the seven source data series. Some country-series were missing completely or had limited time frames of income estimates. For example, neither the WB series nor the IMF series offer estimates for Somalia. Both IMF series for Afghanistan are available only from 2002 to 2015. Some countries showed extremely sparse data coverage, particularly smaller countries, such as Aruba or Turks and Caicos, which only had estimates from the UN series. As noted above, estimates for the constituent republics of the USSR are completely missing for the years prior to the dissolution of the USSR. In our modeling approach, we sought to complete each time series for each country from 1950 to 2015 for a total of 97,020 country-year-series data points. Put another way, our database was missing roughly 50% of its estimates. From the completed database, we intended to generate the two new Institute for Health Metrics and Evaluation (IHME) GDP per capita series.

In the first stage we fill in missing years for existing series by making the assumption that 1) GDP growth rates may vary slightly between sources but that overall there should exist a reliable relationship between a given source’s growth rates and the other sources’ growth rates, and that 2) stability in these predictions could be provided by averaging the results of the independent regressions for each source. We pooled the data from all country-years and estimated the annual growth rates for each country-year for each of the series. We applied ordinary least-squares (OLS) regressions on the exponential growth rates (EGR) of each GDP series as both the outcome and predictor variable, i.e., we regressed the growth rates from each series on those from every other series. For example, to estimate the exponential growth rate for series *k* in year *t* estimated from series *j* in year *t*, we conduct the following model:

(1) $$EGR_{j,k,t}=\beta_{0,j,k}+\beta_{1,j,k}EGR_{j,t},\forall k\neq j$$

This stage yielded six growth rates for each series – one for the relationship between each pair of series. We then averaged these growth rates across the total number of other series to produce an estimated growth rate for each series (same model subscript as above):

(2) $$Predicted\;EGR_{j,k,t}=\frac{1}{L}\sum {}_{j\neq k}EGR_{j,k,t}$$

For an overall example, the exponential growth rate from the UNSTAT estimates were regressed on the growth rates from the IMF ID, IMF USD, Maddison, WB ID, and WB USD, each as separate models. The growth rate predicted from each of these six regressions was then averaged to produce the estimated growth rate for UNSTAT.

Using this averaged growth rate, we make predictions for country-years that are missing. For example, across the seven series, estimates exist in differing time spans for Somalia from 1950 to 2008, but not all series have estimates for every year in that time span. Using the estimated growth rates we forecast and backcast the missing years in each series. When missing years are flanked by years that do have estimates on both sides (for example, if a series is complete for 1950 to 1970 and 1980 to 1990 but is missing estimates from 1970 to 1980), the chain-forward and chain-backward predictions for this period are averaged. If a series is missing completely from a country, no new GDP per capita levels are predicted at this stage.

**Figure 2:**
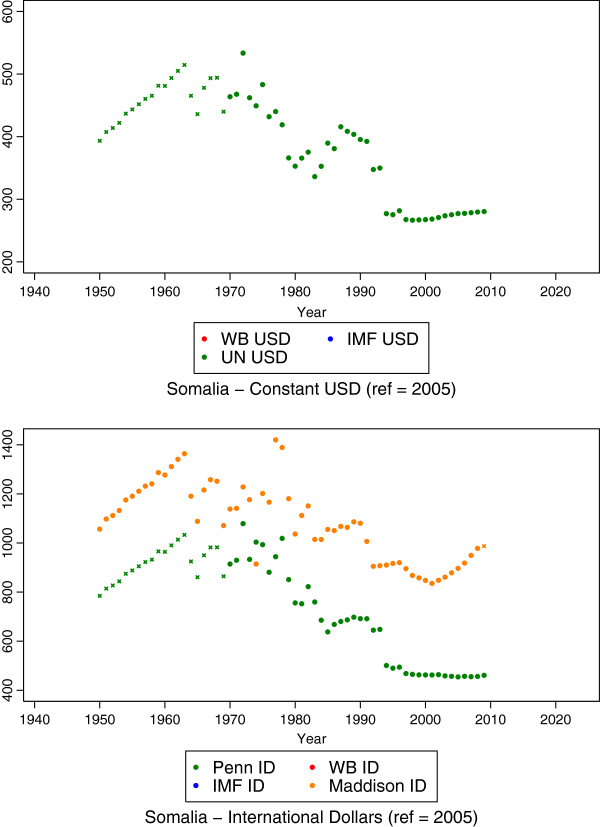
**Results from the second stage estimation for Somalia, which predicts growth rates for each series and then applies the growth rates to chain-forward or chain-backward estimates based on existing data.** Starred data points indicate predictions resulting from this stage.

Coefficients of all regression pairs estimated at this stage were statistically significant with p-values < 0.001 (results not shown). The resulting GDP per capita estimates for Somalia from this stage are shown in Figure 2, where x markers indicate a predicted value.

### Making predictions for a missing series

In the second stage of modeling we generate estimates for series that are completely missing for a given country. For example, the WB ID series does not include estimates for Somalia but does have estimates for 166 other countries. In order to conduct this stage of our model, we made the assumption that the series-to-series relationship that exists in country-years where both series exist should exist in other country-years where only one series provides predictions.

We implemented a mixed-effects model to estimate the relationship between different series. Using data from all country-years combined, we applied the following model for series *i* and country *c* in Global Burden of Diseases, Injuries, and Risk Factors (GBD) Study region *r* being predicted by series *j* for year *t*:

(3) $$In\;GDP\;per\;capita_{i,c,r,t}=\beta_{0}+\beta_{1}\;In\;GDP\;per\;capita_{j,c,r,t}+\alpha_{c,r}+\gamma_{c,r}\;In\;GDP\;per\;capita_{j,c,r,t}$$

where the ln GDP per capita of each series is regressed against all other series, similar to the growth rates stage above. The model includes a country-nested-in-region random intercept $\alpha_{c,r}$ to capture effects that may be intrinsic to a particular region-country. To capture the potential differential relationship between GDP series and countries, we incorporated a country-nested-in-region random slope $\gamma_{c,r}$ on $In\;GDP\;per\;capita_{j,c,r}$. These additional model specifications are based on the assumption that there is an association between a country’s economic patterns and other countries in that region. We also conducted a nonnested version of the same model and observed that the model was not sensitive to the nesting based on the ultimate GDP per capita estimates resulting from each approach. Regions were determined by the GBD Study [29] and are based on both geographical location and economic status.

**Figure 3:**
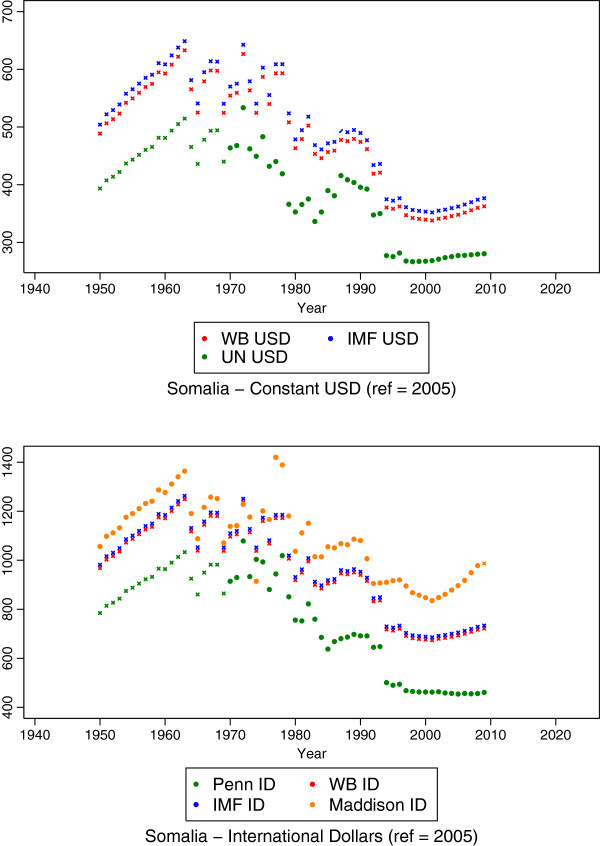
**Estimates resulting from the third stage of the estimation for Somalia, in which a mixed effects model produces estimates for series that are completely missing from a country.** Estimates are indicated by starred data points.

Using the coefficients from the models, we predict estimates for each separate model. For example, we estimate ln GDP per capita of the Penn series for missing country-years as a function of each of the other series as separate models. Then, the ln GDP per capita estimates for Penn predicted by each series are averaged to fill in missing values for Penn for missing country-years. This results in Penn being complete for all country-years for which at least one source has an estimate. For example, Somalia originally had estimates from three series ranging from 1950 to 2009, and after this stage it has estimates for all seven series, complete from 1950 to 2009. The outcome of this stage for the Somalia example is shown in Figure 3, where x markers indicate all predicted values. Coefficients of all regression pairs from these regressions were statistically significant with p <0.001 (results not shown).

### Making out-of-sample predictions

In the third stage, we fill in all missing years between 1950 and 2015 for all countries. In Somalia, across the series there exist estimates from 1950 to 2009, but no estimates for the period from 2009 to 2015. To estimate the GDP growth in this period, we wanted to capture the spatial-temporal growth trends suggested by general growth trends in those years and by countries in the same region that had more complete data. We used a mixed effects model, where the dependent variable was the exponential growth rate of GDP per capita for each country *c*, each series *j,* and all years *t* ($EGR_{c,j,t}$), i.e., in this model we pooled data from all the series-country-years into one model. The model included fixed effects for indicator (i.e., 0 or 1) variables for each data source, a country-nested-in-region random effect, $\alpha_{r,c}$, and a random effect on year, $\delta_{t}$. These model specifications reflect our assumption that in the case of complete data sparseness for a country-year, the economic trends occurring in other countries in the same region and at the same time can approximate what will be occurring in the country-year with no data. By modeling growth rates instead of GDP levels at this stage, we are also able to prevent any discontinuities due to data coverage. The model form is provided below, where each source’s specification is the indicator variable for that source. For example, $\beta_{IMF\;ID}IMF\;ID$ is the coefficient and variable for a variable that is a 0 for values not in the IMF ID series and a 1 for values in the IMF ID series.

(4) $$EGR_{c,j,t}=\beta_{0}+\beta_{IMF\;ID}IMF\;ID+\beta_{IMF\;USD}IMF\;USD+\beta_{\mathrm{Penn}}Penn+\beta_{\mathrm{UNSTAT}}UNSTAT+\beta_{WB\;ID}WB\;ID+\beta_{WB\;USD}WB\;USD+\delta_{t}+\alpha_{r,c}$$

**Figure 4:**
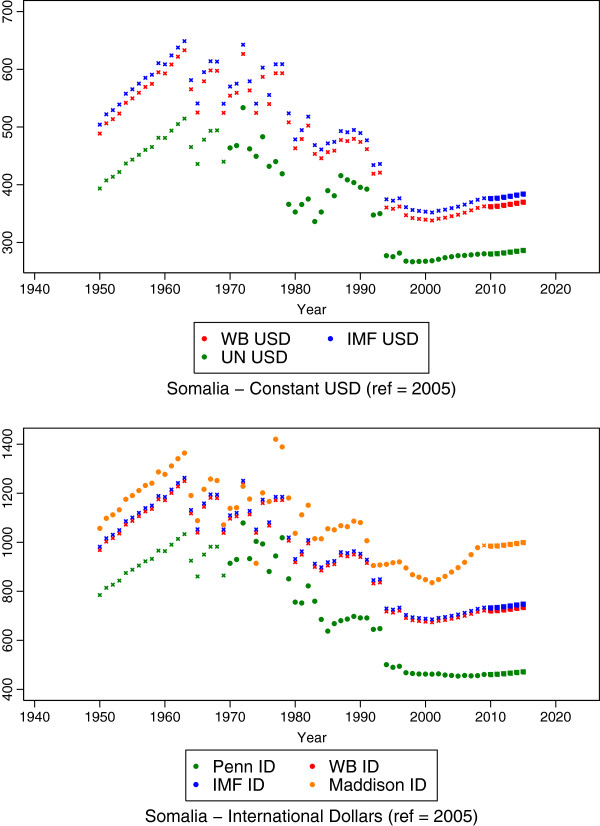
**GDP per capita estimates resulting from the final stage of the model for Somalia, in which a mixed effects model predicts out-of-sample data points for each country.** Somalia was missing data after 2009, and this stage created predictions through 2015 based on year random effects and country-nested-in-region random effects. Bolded square data points indicate estimations made at this stage and starred data points indicate predictions from previous stages.

This regression produces estimates of growth rates for all missing series-country-years. These growth rates were applied to existing GDP per capita levels to forecast or backcast estimates in order to complete each series for each country from 1950 to 2015. The resulting estimates from this stage for Somalia are shown in Figure 4, where squares indicate the predicted values from this stage.

### Creating estimates for former USSR republics

One of the myriad complications in estimating and analyzing a comprehensive time series is approaching the changes in countries’ sovereignty status. It is difficult to produce a time series that is both comprehensive and appropriately reflective of geopolitical chronology. The USSR republics posed a unique challenge in our estimation process. None of the data sources had estimates for any of the constituent republics (Armenia, Azerbaijan, Belarus, Estonia, Georgia, Kazakhstan, Kyrgyzstan, Latvia, Lithuania, Moldova, Russia, Tajikistan, Turkmenistan, Ukraine, Uzbekistan) prior to 1990. We attempted to include these republics in the estimation process described above, but the model tended to predict very low or nonexistent growth rates for the period from 1950 to 1990, which did not seem feasible considering the aggressive economic growth of the Soviet Union during certain periods of this era [30]. We concluded that our approach needed slight modification to be used successfully with the USSR republics and conducted the following steps (which roughly follow our general approach and hold similar assumptions as specified above) at an early stage of our overall modeling process.

We made the additional assumption that there should be a relationship between the economic growth of each of the USSR republics and the USSR itself with the caveat that this association would be different between each constituent republic and the USSR. Based on this assumption, we developed a method to predict for this missing time span for each constituent republic. Our method is structured similarly to the estimation process described for the other countries above with some modifications. One source, Maddison, produced historical estimates for the former USSR itself (ranging from 1950 to 2008), as well as for the constituent republics from 1990 to 2008. We applied separate OLS regressions for each USSR republic to predict the ln GDP per capita from 1950 to 1990 for that republic *c* in the Maddison series dependent on the Maddison estimates for the USSR itself:

(5) $$In\;GDP\;per\;capita_{Maddison,c}=\beta_{0}+\beta_{1}\;In\;GDP\;per\;capita_{Maddison,Former\;USSR}$$

This stage is intended to capture the relationship between the USSR and a given republic and is expected to be slightly different for each republic. This stage yielded estimates for the Maddison series for each USSR republic from 1950 to 2008. We then added the other series’ estimates for each of the USSR republics, which were typically complete from 1990 to between 2008 and 2015. We predicted the GDP per capita EGRs for the missing years for series *s* by conducting a mixed effects model of each series’ growth rates dependent on Maddison’s growth rates with a random effect $\alpha_{c}$ on republic:

(6) $$EGR_{s}=\beta_{0}+\beta_{1}EGR_{\mathrm{Maddison}}+\alpha_{c}$$

The final stage is to produce estimates for any missing series for a particular republic. We used a similar model as the second stage described above, with a random intercept $\alpha_{c}$ for each constituent republic and a random slope $\beta_{1}$ on series:

(7) $$In\;GDP\;per\;capita_{i,c,t}=\beta_{0}+\beta_{1}\;In\;GDP\;per\;capita_{j,c,t}+\alpha_{c}+\gamma_{c}\;In\;GDP\;per\;capita_{j,c,t}$$

Following this step, the USSR estimates were added to the global estimates prior to the third stage of the modeling process described above. Thus, all USSR countries were a part of the final mixed effects model that ensured all series and countries were complete from 1950 to 2015.

Cumulatively the steps described above allowed us to achieve Goal 1 of our study.

### Derivation of a new GDP per capita series

**Figure 5:**
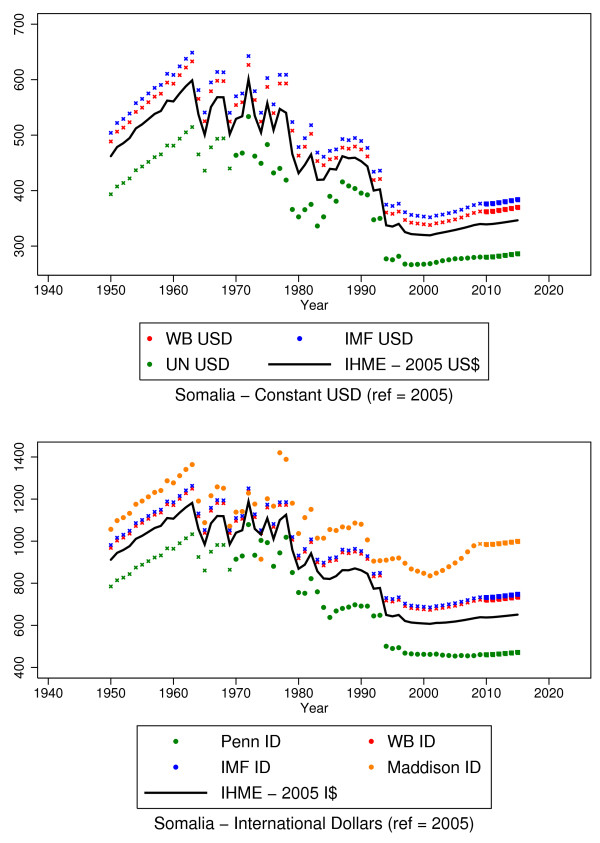
**The individual source series resulting from all estimations and the IHME series, which are produced from averaging the source series.** Note that the Maddison series is not used in generating the IHME ID series since it uses a different base year.

As explained above, Goal 2 of our study was to create new GDP per capita series in order to address the systematic differences observed between series. For example, Figure 4 shows that even with the completed time series for Somalia, the different sources produce very different estimates, even though the trends are similar. For a given year, e.g., 2011, the estimates of GDP per capita in ID for Somalia range from $461 in the Penn ID series to $734 in the IMF ID series. In the USD estimates for Somalia in 2011, the estimates range from $281 in the UNSTAT series to $377 in the IMF USD series. In the absence of objective criteria that can be used to determine which series is more appropriate to use, we created a new set of estimates for both the constant 2005 USD series and for the 2005 ID series by averaging estimates from each of the three constituent data series. As noted previously, the Maddison series was excluded from creating these series due to its different base year. These two new series are shown for Somalia in comparison to the other series in Figure 5. This stage allowed us to accomplish Goal 2 of our study.

### Exploring the sensitivity of results to the choice of series

One premise of this project was the idea that the use of different income series could affect the outcomes and inferences drawn from a statistical model that uses income as a predictor or covariate. We investigated this question by conducting regressions to model under-5 mortality (5q0) and adult male and female mortality (45q15). Specifically, we conducted a first-differences model with these health outcomes as the dependent variable and income, female education, and HIV seroprevalence (three-year lag) as the independent variables. Separately, we also ran a Beck and Katz model that in addition to the independent variables also uses a one-year lag of the outcome variable as a predictor variable. For the income covariate, we used each of the seven original income series and then each of the nine complete income series (including the two IHME series). All outcome and predictor variables were modeled in log space.

### Analysis details

Stata 11.0 was used for all analysis and data management. All data and code are available from the authors upon request.

## Results

GDP per capita estimates for 210 countries from 1950 to 2015 are provided in Additional file 3: Annex 3. The estimates are provided for each of the seven source series used for analysis (IMF ID, Penn ID, World Bank ID, Maddison ID, World Bank USD, IMF USD, and UNSTAT USD) and for the new IHME ID and USD series. The USD estimates are all expressed in constant 2005 USD terms, as are all of the ID (PPP) series except for Maddison, which uses 1990 as a base year. These series are the most comprehensive GDP per capita series currently available and offer researchers diverse options to serve different analytical purposes.

Table 3 shows the results from the analysis where we explored whether the different series yield different coefficients for the relationship between income per capita and under-5 and adult mortality. Results from the Maddison regression are not shown since the Maddison series is expressed in a different base year than the other series, and the purpose of this analysis was solely to analyze interseries differences. Table 3 shows that there is notable interseries variation in using the original data series and that using the completed series attenuates the interseries variation in the outcome coefficient. For example, in the first-differences model with under-5 mortality as the dependent variable, the range of the coefficient on GDP across series is much greater in the original data series than the completed ones. Even more importantly, within the USD series and the ID series, the estimated coefficients on GDP per capita differ significantly across the three original series, indicating that the choice of series will significantly affect conclusions drawn about the relationship between GDP and under-5 mortality. However, using the completed data series removes this problem. The same finding is observed for adult mortality as well, where using the original data series produces significantly different coefficients for GDP per capita across the series. These results suggest that the development of complete income series is important not only because it allows for more comprehensive modeling, but also because using complete income series mitigates variation in quantitative inferences.

**Table 3:** Example of series effect on health outcome modeling

	**Series**	**Child mortality (5q0)**	**First-differences**	
			**Adult mortality, female (45q15)**	**Adult mortality, male (45q15)**
Original series	IMF ID	-0.480 (-0.495, -0.464)	-0.286 (-0.299, -0.273)	-0.278 (-0.289, -0.266)
	Penn ID	-0.389 (-0.402, -0.377)	-0.252 (-0.262, -0.242)	-0.235 (-0.244, -0.226)
	WB ID	-0.490 (-0.505, -0.474)	-0.288 (-0.301, -0.276)	-0.273 (-0.285, -0.261)
	WB USD	-0.331 (-0.342, -0.321)	-0.200 (-0.208, -0.192)	-0.185 (-0.193, -0.178)
	IMF USD	-0.378 (-0.390, -0.367)	-0.215 (-0.225, -0.205)	-0.214 (-0.223, -0.205)
	UNSTAT USD	-0.306 (-0.315, -0.297)	-0.185 (-0.193, -0.178)	-0.173 (-0.180, -0.166)
Completed series	IMF ID	-0.390 (-0.402, -0.378)	-0.244 (-0.253, -0.235)	-0.227 (-0.235, -0.219)
	Penn ID	-0.371 (-0.383, -0.359)	-0.239 (-0.248, -0.231)	-0.224 (-0.232, -0.216)
	WB ID	-0.377 (-0.389, -0.365)	-0.239 (-0.248, -0.230)	-0.222 (-0.230, -0.214)
	WB USD	-0.324 (-0.333, -0.315)	-0.188 (-0.195, -0.181)	-0.178 (-0.185, -0.172)
	IMF USD	-0.324 (-0.333, -0.315)	-0.189 (-0.196, -0.182)	-0.179 (-0.186, -0.173)
	UNSTAT USD	-0.320 (-0.330, -0.311)	-0.185 (-0.192, -0.178)	-0.175 (-0.182, -0.169)
	IHME ID	-0.386 (-0.398, -0.374)	-0.245 (-0.254, -0.236)	-0.229 (-0.237, -0.221)
	IHME USD	-0.324 (-0.334, -0.315)	-0.189 (-0.196, -0.182)	-0.179 (-0.185, -0.172)
	**Series**	**Child mortality (5q0)**	**Beck and Katz**	
			**Adult mortality, female (45q15)**	**Adult mortality, male (45q15)**
Original series	IMF ID	-0.507 (-0.524, -0.491)	-0.307 (-0.321, -0.293)	-0.287 (-0.300, -0.274)
	Penn ID	-0.399 (-0.413, -0.386)	-0.272 (-0.283, -0.262)	-0.244 (-0.254, -0.234)
	WB ID	-0.509 (-0.526, -0.492)	-0.303 (-0.316, -0.289)	-0.282 (-0.295, -0.269)
	WB USD	-0.347 (-0.358, -0.336)	-0.213 (-0.222, -0.204)	-0.194 (-0.203, -0.186)
	IMF USD	-0.403 (-0.416, -0.391)	-0.233 (-0.244, -0.222)	-0.223 (-0.233, -0.213)
	UNSTAT USD	-0.326 (-0.336, -0.315)	-0.201 (-0.209, -0.192)	-0.183 (-0.191, -0.176)
Completed series	IMF ID	-0.412 (-0.426, -0.399)	-0.262 (-0.272, -0.252)	-0.239 (-0.249, -0.230)
	Penn ID	-0.390 (-0.403, -0.377)	-0.260 (-0.269, -0.250)	-0.238 (-0.247, -0.229)
	WB ID	-0.397 (-0.411, -0.384)	-0.256 (-0.265, -0.246)	-0.234 (-0.243, -0.225)
	WB USD	-0.344 (-0.354, -0.333)	-0.202 (-0.210, -0.194)	-0.189 (-0.196, -0.181)
	IMF USD	-0.344 (-0.354, -0.334)	-0.204 (-0.212, -0.196)	-0.190 (-0.197, -0.183)
	UNSTAT USD	-0.338 (-0.349, -0.328)	-0.199 (-0.206, -0.191)	-0.185 (-0.192, -0.177)
	IHME ID	-0.408 (-0.421, -0.395)	-0.264 (-0.274, -0.255)	-0.242 (-0.251, -0.233)
	IHME USD	-0.344 (-0.354, -0.334)	-0.203 (-0.211, -0.195)	-0.189 (-0.196, -0.182)

We modeled under-5 mortality, adult female mortality, and adult male mortality from 1970 to 2010 as a function of log income from each of the different series, both in their original forms and completed forms. The Beck and Katz model includes a one-year lag of the outcome variable as a predictor variable. Both models control for female educational attainment and a three-year lag on HIV seroprevalence.

## Discussion

In this paper we have proposed and accomplished our two goals of 1) providing a method for producing a complete time series for GDP per capita for all existing sources and 2) proposing a new series to be used as an alternative to the existing series. By accomplishing these two goals we have produced a resource that will be useful for myriad purposes. Each of the source data series is now usable for 210 countries from 1950 to 2015. The strength of the IHME data series is that they reduce the bias that may result from using one source’s series for analyses. Assumptions, methods, and data availability may differ from source to source [26,31], and it is not clear whether one source’s methods are superior to that of any other. Some researchers may have a deliberate reason to use one particular series and they will no longer be limited by data availability.

The first goal of our study was designed to address the fact that population health analyses were limited previously by spatial or temporal limitations of the existing data. Our method of completing the time series preserves series-specific trends and nuances throughout the estimation process and creates series that extend the existing data to missing countries and years. This should facilitate population health analyses and reduce biases that arise from using series with missing country-years [32,33]. To illustrate this, we provided an example of the type of bias that can arise in missing time series values in our analysis of different GDP series in their original versus imputed state as predictor variables for mortality and indicate how bias can be reduced through the use of a more complete time series. We note that the estimates for GDP for post-2010 are driven solely by the growth estimated by the IMF series and that speculation on future years’ GDP growth should be interpreted and used cautiously. Similarly, the estimates for GDP per capita for emerging economies are more subject to variation between different series, as is indicated in Figure 1, and to higher uncertainty. This caveat is important in the use and interpretation of any GDP series for emerging economies.

In imputing these series, we sought to retain as much flexibility in the usage of the data series as possible. The inclusion of both constant USD series and PPP-based ID series offers such flexibility. The ID series controls for the idiosyncratic qualities of a country’s economy that affect the cost of goods for consumers. For example, due to economic policy, agriculture, and geography, a particular quantity of food may cost much more in one country than another. The pathways through which income affects health and other social outcomes may relate to the goods and services that can be afforded at different incomes. As such, we recommend the use of the IHME ID series for users who are modeling a health or social outcome affected by individual behavior or opportunity and who wish to control for income or to use income as a covariate.

In contrast, the USD series are derived entirely from the empirical amounts of trade occurring in a country without taking into consideration the cost of goods to consumers. Consequently, the IHME USD series (or any of the other USD series) may be a better option for researchers interested in exploring finance, trade, government spending, or other econometric topics that involve the movement of fungible assets.

Regardless of whether researchers opt to use a USD or ID series, we recommend that researchers test the sensitivity of their findings to using alternative completed income series. This task has also been made much more accessible since each series is provided in exactly the same format, whereas previously it was difficult to switch from one series to another due to formatting and naming nuances.

As additional analysis beyond our two research goals in this paper, we explored the effects that using different income time series could have on a statistical model of a health outcome. Using under-5 and adult mortality as exemplary health outcomes, we found that completed income time series could be interchanged without significantly affecting the regression’s income coefficients. Nevertheless, researchers should test the sensitivity of any income-dependent analysis using each of the different completed income series.

The role of income as a driver of health means that it can serve a wide array of analytical purposes. Previous analyses and studies that invoked econometrics may have been limited by the availability of income estimates for different countries and years. Thus, the completion of existing GDP per capita series and the development of the new IHME GDP per capita series provide useful resources for economic, demographic, and population health research. We have included all existing major data sources in this project, and our proposed modeling framework allows for easy updating of estimates when each of the sources updates its series.

## Abbreviations

GDP, Gross domestic product; IHME, Institute for Health Metrics and Evaluation; ID, International dollars converted to purchasing power parity terms; LCU, Local currency units; USD, United States dollars; PPP, Purchasing power parity; Penn, University of Pennsylvania Center for International Comparisons of Production; Maddison, Angus Maddison’s research homepage at the University of Groningen Department of Economics; IMF, International Monetary Fund; abbreviation refers to World Economic Outlook report; UNSTAT, United Nations Statistics Division; abbreviation refers to National Accounts Main Aggregates Database; WB, World Bank; abbreviation refers to World Development Indicators.

## Competing interests

The authors declare that they have no competing interests.

## Authors' contributions

SLJ prepared data, developed the model, and drafted the paper. PG prepared data and developed early stages of the model. CJLM guided the model development and provided insight on econometric analysis. EG guided the model development and edited the paper. All authors read and approved the final manuscript.

## Supplementary Material

Additional file 1: Annex 1 GDP per capita estimates for Somalia that result from each stage of the modeling process. Click here for file

Additional file 2: Annex 2 List of outliers dropped from original data sources based on expert opinion. Click here for file

Additional file 3: Annex 3 GDP per capita estimates for seven source series and two IHME series and indicators for each estimate from the original data or from our imputation. Click here for file
